# Understanding microbiome dynamics via interpretable graph representation learning

**DOI:** 10.1038/s41598-023-29098-7

**Published:** 2023-02-04

**Authors:** Kateryna Melnyk, Kuba Weimann, Tim O. F. Conrad

**Affiliations:** 1 Department of Mathematics and Computer Science, Freie Universität Berlin, Arnimallee 6, 14195 Berlin, Germany; 2 Zuse Institute Berlin, Takustraße 7, 14195 Berlin, Germany

**Keywords:** Microbiology, Applied mathematics, Computer science

## Abstract

Large-scale perturbations in the microbiome constitution are strongly correlated, whether as a driver or a consequence, with the health and functioning of human physiology. However, understanding the difference in the microbiome profiles of healthy and ill individuals can be complicated due to the large number of complex interactions among microbes. We propose to model these interactions as a time-evolving graph where nodes represent microbes and edges are interactions among them. Motivated by the need to analyse such complex interactions, we develop a method that can learn a low-dimensional representation of the time-evolving graph while maintaining the dynamics occurring in the high-dimensional space. Through our experiments, we show that we can extract graph features such as clusters of nodes or edges that have the highest impact on the model to learn the low-dimensional representation. This information is crucial for identifying microbes and interactions among them that are strongly correlated with clinical diseases. We conduct our experiments on both synthetic and real-world microbiome datasets.

## Introduction

Complex microbiome ecosystems have a strong impact on the health and functioning of human physiology. Large-scale perturbations in the microbiome constitution are strongly correlated, whether as a driver or a consequence, with clinical diseases, such as inflammatory bowel disease^1,2^, obesity^3^, and some types of cancer^4–7^.

Many studies have been aimed at accurately differentiating the disease state and at understanding the difference in the microbiome profiles of healthy and ill individuals^8,9^. However, most of them mainly focus on various statistical approaches, omitting microbe-microbe interactions between a large number of microbiome species that, in principle, drive microbiome dynamics. In addition, some studies make use of the concept of a potential landscape in physics^10–12^, giving completely new insight into the analysis of microbiome dynamics. Namely, a healthy human microbiome can be considered as a metastable state lying in a minimum of some potential landscape. The system of time-evolving interactions of species appears to be equilibrated for a short timescale but at larger timescales a disease or other strongly impacting factors, such as antibiotic exposure, makes the system undergo transitions from one metastable state (healthy) to other metastable states (diseased).

Detecting metastable states and associated interactions of species, which undergo changes from one metastable state to others, is complicated by the high dimensionality and the compositional nature of microbiome data. Therefore, we propose a method that simplifies the analysis and prediction of the large-scale dynamics of microbiome composition by projecting this system onto a low-dimensional space. First, to allow interactions between species to change over time, we represent the system as a time-evolving graph with nodes being microbes and edges being interactions between microbes. Second, we define two key components of our method: (1) the Transformer^13^ that learns both structural patterns of the time-evolving graph and temporal changes of the microbiome system, and (2) contrastive learning that makes the model maintain metastability in a low-dimensional space. To assess the performance of our method, we apply it to the synthetic data from Melnyk et al.^14^, which has known underlying dynamics, and to two real-world microbiome datasets, i.e. MovingPic^9^ and Cholera Infection^15^. Furthermore, we will show that it is feasible to extract topological features of the time-evolving graph which are associated with metastable states and have the highest impact on how the model learns the low-dimensional representation of the time-evolving graph with metastability. This information can help in differentiating the microbiome profile of healthy and diseased individuals.

Our main contribution is presenting a model that learns a low-dimensional representation of the time-evolving graph with metastable behaviour in an unsupervised manner. We show in experiments that the metastability governing the time-evolving graph is preserved by the model. By interpreting the output of the model with respect to the input, we demonstrate that it is feasible to extract topological features of the time-evolving graph, which define each metastable state. These features can be used to identify a set of microbes that drive the microbiome constitution to undergo transitions from one metastable state to others.

## Related work

We can broadly categorize methods for graph representation learning into semi-supervised or unsupervised methods and methods for static or time-evolving (dynamic) graphs. A good overview of the current state of methods for time-evolving and for static graph representation techniques can be found in (Kazemi et al.^16^, Barros et al.^17^ and Cui et al.^18^, Zhang et al.^19^), respectively. The most recent survey on both time-evolving graphs and static graphs is presented in Khoshraftar et al.^20^.

### Static graph representation

Approaches for static graph representations can be classified into two categories – those which learn the representation of nodes and those which learn the representation of sub-structures of the graphs. The first category tends to encode nodes of the graph in a low-dimensional space such that their topological properties are reflected in the new space (node2vec^21^, DeepWalk^22^). Most studies are focused on node representation learning, and only a few learn the representation of the whole graph (graph2vec^23^).

Representing an entire graph using node embeddings is a challenging task because pooling a graph into a vector representation usually introduces an extreme information bottleneck. Simple approaches to this problem aggregate all node embeddings (e.g., sum or average) or create a “master node” that is connected to all the other nodes in the graph. Recent graph pooling operations can learn hierarchical representations that greatly reduce the size of a graph. For instance, the authors in Ying et al.^24^ propose a differentiable graph pooling module called DiffPool that learns to assign nodes to clusters, resulting in a gradual pooling of the graph. Further improvements to hierarchical pooling can potentially reduce the number of parameters, the complexity of the operator, and they might increase the overall performance. For instance, SAGPool^25^ proposes the self-attention mechanism using graph convolution in the graph pooling, and HGP-SL^26^ introduces a structure learning mechanism. In contrast to these approaches, we leverage the self-attention mechanism of the Transformer^13^ in our model and we add a master node that attends to every node in the graph. This simple graph pooling only marginally increases the number of parameters and the computational complexity of the model. Furthermore, we use the initial representation of the master node to inject the topological information of the time-snapshot graph at a previous time step.

### Dynamic graph representation

Representing a time-evolving graph in a low-dimensional space is an emerging topic that is still being investigated. Among recent approaches, DynGEM^27^ uses the learned representation from the previous time step to initialize the current time step representation. Such initialization keeps the representation at the current time step close to the learned representation at the previous time step. The extension of the previous method is dyngraph2vec^28^, where authors have made it possible to choose the number of previous time steps that are used to learn the representation at the next time step. Moreover, dyngraph2vec uses recurrent layers to learn the temporal transitions in the graph. Unlike this method, we utilize the multi-head attention mechanism^13^ to capture the temporal changes in the time-evolving graph.

Another category of methods that are successful in graph representation learning is Graph Neural Networks. One of the methods is EvolveGCN^29^ which applies the graph neural network for static graphs to dynamic graphs by introducing a recurrent mechanism to update the network parameters. The authors focus on the graph convolutional network and incorporate a recurrent neural network to capture the dynamics of the graph. Recently, attention-based methods have been extensively proposed. One of them is DynSAT^30^ which learns a dynamic node representation by considering topological structure (neighbourhood) and historical representations following the self-attention mechanism. However, one of the disadvantages of these methods for our problem is that time-evolving graphs with metastability usually consist of many time steps, which results in the increase of time that is needed to learn a low-dimensional representation. Another disadvantage is that all these methods capture the dynamic of nodes and, as a result, output the low-dimensional representation of nodes.

## Results

### Datasets

Here, we briefly describe the datasets used to evaluate the model. Besides experiments with synthetic datasets, we show the application of our method to real-world microbiome data. Both the idea of generating synthetic datasets and the idea of pre-processing real-world datasets are explained in more detail in Melnyk et al.^14^. An overview of the datasets used in this paper is shown in Table 1.

**Table 1:** Statistics of each dataset used in this paper.

Name	# Nodes	# Edges (avg.)	# Time steps	# States
npos_2WellGraph	150	10821	10000	2
pos_2WellGraph	100	4109	10000	2
pos_3WellGraph	150	10869	10000	3
CholeraInf	96	106	34	2
MovingPic	919	10602	658	2

#### Synthetic data

To estimate how the proposed method can capture the dynamics of the time-evolving graph and learn a proper low-dimensional representation, we generate synthetic datasets with known topological and temporal patterns (for more details see Melnyk et al.^14^). We make use of a molecular dynamics inspired problem, namely diffusion in a two-dimensional energy landscape given by the following stochastic differential equation

1 $$\begin{aligned} dX_t = -\nabla V(X_t)dt + \sqrt{2 \beta ^{-1}}dW_t \end{aligned}$$

with the potential function:

2 $$\begin{aligned} V(x) = \cos (s \arctan (x_2, x_1)) + 10\Big (\sqrt{x_1^2 + x_2^2} - 1\Big )^2, \end{aligned}$$

where *s* is the number of metastable states or wells, $W_t$ is a standard Wiener process, $\beta$ is the inverse temperature that controls the transition between states. The higher $\beta$, the less likely the transition from one state to another is. We use the potential function (2) with $s=3$ to generate a time-evolving graph with three metastable states (pos_3WellGraph). For the synthetic datasets with 2 metastable states, pos_2WellGraph and npos_2WellGraph, we use the following potential function, which is often called a double-well potential:

3 $$\begin{aligned} V(x) = \frac{x^4}{4} - \frac{x^2}{2}. \end{aligned}$$

To construct a time-evolving graph $\mathbb {G} = \{G_1, \dots G_{T}\}$, one trajectory $S = \{(x^i_1, x^i_2)\}_{i=1}^T$ is generated using SDE 1. Then, a fully-connected graph is defined with the number of nodes *n* and each node has a coordinate $(a_j, b_j), j=1, \dots , n$. For each *t*, we draw a circle with a centre in the point $(x_1^{(t)}, x_2^{(t)})$ and with a pre-defined radius *r*. We then remove all edges between vertices that are inside the circle at time *t*. In order to add noise to the data we randomly remove edges outside the circle as well.

We further split our synthetic datasets into two categories: positional and non-positional data. Positional data means that we use the positional encoding (see Sect. “Methods” below for details) before training the model. Non-positional data means that we do not use positional encoding, as the topological structure of the time-evolving graph can be understood without providing the positional information of the node. We define these two categories to empirically show that our model does not rely on the positions of nodes if the topological patterns of each state are clearly defined.

##### Positional data

This synthetic data has a topological structure that is difficult to distinguish without the positions of nodes. We briefly describe the three-step process, which generates the positional time-evolving graph:

- We sample the trajectory $\mathscr {S} = \{(x_1^{(i)}, x_2^{(i)})\}_{i=1}^{T}$ using SDE (1), and the corresponding potential function (2) or (3).
- Then we choose the number of nodes *n*, and assign random coordinates $(a_j, b_j), j=1, \dots , n$ to each of these nodes. This is done because we need to know the locations of discriminating features of the metastable states.
- In the final step, we define discriminating topological features for each state. Let $G_0$ be a complete graph. In the case of the *s*-well potential, we generate $G_t$, $\forall t, t = 1, \dots , T$ by drawing a circle with the centre at $(x_1^t, x_2^t)$ and the radius *r* and randomly removing edges between nodes that are inside the current circle. In addition, to address the noise in the real-world data, we also remove edges outside the current circle. For double-well potential, we remove edges between nodes, which satisfy $b_j > \frac{1}{T} \sum _{i=1}^T x_1^i$.

**Figure 1:**
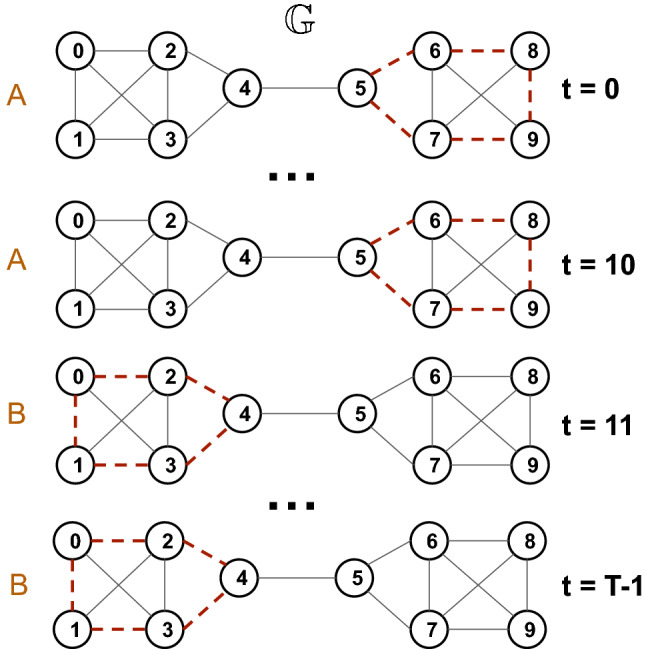
The example of a time-evolving graph with metastability where two states *A* and *B* are difficult to distinguish since they are topologically the same. Red dashed edges are removed from the time-evolving graph $\mathbb {G}$.

We can see that the graph features of different states are difficult to distinguish, and the model will fail to discriminate between metastable states in the time-evolving graph. As an illustration of that, we provide Fig. 1. There are two states *A* and *B* of the time-evolving graph $\mathbb {G}$ that are characterized by removed edges in the right part of $\mathbb {G}$ for the state A and in the left part of the graph for state B. Topologically, states have the same neighbourhood structures, which will result in the same points in the low-dimensional space. The same occurs for our synthetic dataset: nodes in the circles determine metastable states, and the neighbourhoods of these nodes are almost identical for the model.

##### Non-positional data

This type of data has a dissimilar topological pattern to the positional data. The time-evolving graph is generated in the same way as the positional synthetic dataset, except instead of removing a random number of edges between nodes that fall in the circle, we remove edges between nodes in the circle in such a way that each node has a particular number of neighbours. We define the number of removed neighbours of nodes arbitrarily and differently for each state.

#### Real-world dataset

##### MovingPic

This dataset, originally introduced in Caporaso et al.^9^, is the first real-world dataset on which we evaluate our model. In this study, one male and one female were sampled daily at three body sites (gut, skin, and mouth) for 15 months and for 6 months, respectively. To obtain a time-evolving graph, we pre-process Operational Taxonomic Units (OTU) that contain the number of 16S rDNA marker gene sequences that are observed for each taxonomic unit in each sample. Let $D \in \mathbb {R}^{T \times p}$ be an OTU table, where *T* is the number of time points and *p* is the number of OTUs. As this data does not have any obvious perturbations, such as antibiotics exposure or diseases, which could potentially create a metastable structure, an artificial noisy signal is added to the data. The Pearson correlation between two OTUs is computed, and then the initial time-snapshot graph is constructed. To construct time-snapshot graphs at each time step, we have used the OTU table to remove edges between nodes^14^. If the OTU count for a particular node is zero, then the edge is removed between this node and its neighbouring nodes.

##### CholeraInf

This dataset has been introduced in a study about the recovery from Vibrio Cholera infection^15^. Here, faecal microbiota was collected from seven cholera patients from disease (state 1) through recovery (state 2) periods. Moreover, in our experiment, we use the microbiome of one patient, since the variation in the microbiome constitution among individuals can have an impact on the result of the model. The time-evolving graph is obtained in the same way as it has been done for the MovingPic dataset.

### Visualization and comparative analysis

In this part, we focus on verifying the qualitative performance of our model. As a first experiment, we visualize the resulting graph embedding to evaluate how separated the metastable states are in the low-dimensional space. As a second experiment, we compare our model with the following methods: a simple baseline chosen from state-of-the-art methods for dimensional reduction, namely, Principal Component Analysis (PCA), two kernel-based methods graphKKE^14^ and WL kernel^31^, and two graph representation learning methods node2vec^21^ and graph2vec^23^.

- PCA is a method for dimensional reduction. To be able to apply this method to the time-evolving graph, we flatten an adjacency matrix of each time-snapshot graph into a vector.
- The graphKKE approach is proposed for learning the embedding of a time-evolving graph with metastability. It is a graph kernel-based method that combines a transfer operator theory and a graph kernel technique.
- The WL kernel decomposes graphs into rooted subgraphs using a relabelling process and computes feature vectors based on the number of initial and updated labels.
- The graph2vec approach projects the set of static graphs, and it comprises two main components: (1) Weisfeiler– Lehman relabeling process and (2) the skip-gram procedure from doc2vec^32^.
- The node2vec algorithm is a node representation method that uses breadth-first search and depth-first search to extract local and global information from the static graph.

#### Evaluation metric

In order to conduct the comparison analysis, we use a standard clustering evaluation metric — Adjusted Rand Index(ARI). The ARI values lie in the range $[-1;1]$ with 0 representing random clustering and 1 being the highest correspondence to the ground-truth data. We report ARI on the test set for all datasets.

#### Experimental setup

First, we examine the evolution of the graph embedding by visualizing it at the beginning, in the middle and at the end of the training of the model. To do so, we use the graph embedding $\hat{g} =\{\hat{g}_1, \dots , \hat{g}_{T}\}$, where $\hat{g}_i \in \mathbb {R}^{d}$ with $d=2$. For all synthetic datasets, we set the hyperparameter *l* to be 3, the batch size to be 64, and the number of epochs to be 200 for both pos_3WellGraph and npos_2WellGraph. For real-world data, the batch size is set to 64 for MovingPic and 6 for the CholeraInf dataset. We use the Adam optimizer with default parameters, the number of heads of the Transformer is 4 and the number of layers in the Transformer is 3. The temperature parameter of the contrastive loss is 1 for all datasets. As the graphKKE method approximates the eigenfunctions of a transfer operator, the dimension of the graph embedding equals the number of metastable states in the time-evolving graph. This means that we need to apply a dimensional reduction method to be able to visualize it. Thus, PCA is applied to the output of graphKKE with the number of components to be 2. We also apply PCA to the flattened adjacency matrices with the number of components to be 2. Moreover, since we are interested in whether metastable states of the original space correspond to the clusters of points in the reduced space, the points of the graph embeddings are coloured according to the original ground truth metastable states.

For the comparison analysis, we obtain graph embeddings from other methods in the following way. We set the number of dimensions of graph embeddings to 32 for our method, node2vec, graph2vec and PCA. We use the implementations for node2vec and graph2vec with the default hyperparameters provided by the authors. In the graphKKE method, the number of dimensions of the final graph embedding equals the number of metastable states in the time-evolving graph. Finally, we apply the *k*-means method to cluster points of the final graph embeddings of each method. However, node2vec is developed to learn node representations, which is why, to obtain embeddings of the entire time-snapshots graph, we average node embeddings of each time-snapshot graph.

#### Result and discussion: synthetic data

**Figure 2:**
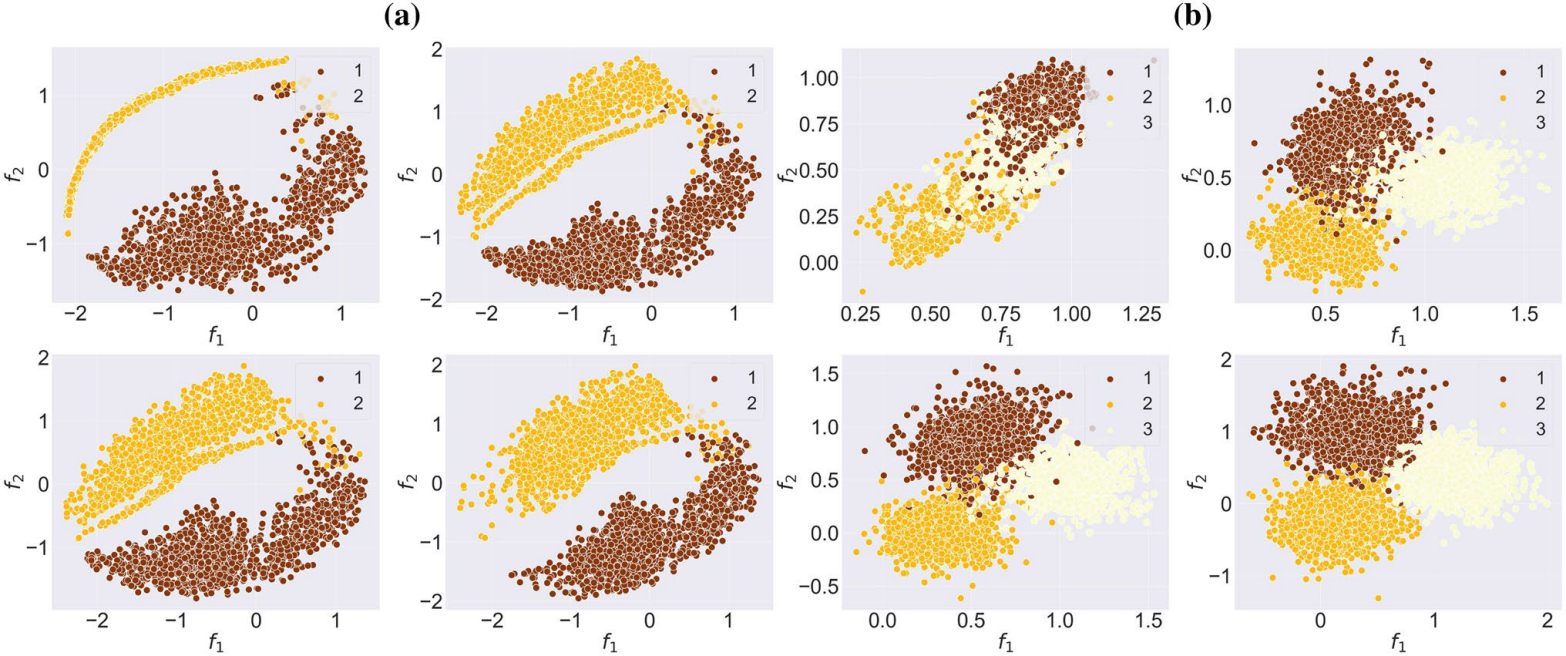
The evolution of the graph embedding of the time-evolving graph $\mathbb {G}$ during the training of our model on (**a**) npos_2WellGraph and (**b**) pos_3WellGraph. The points are coloured according to ground-truth labels.

**Figure 3:**
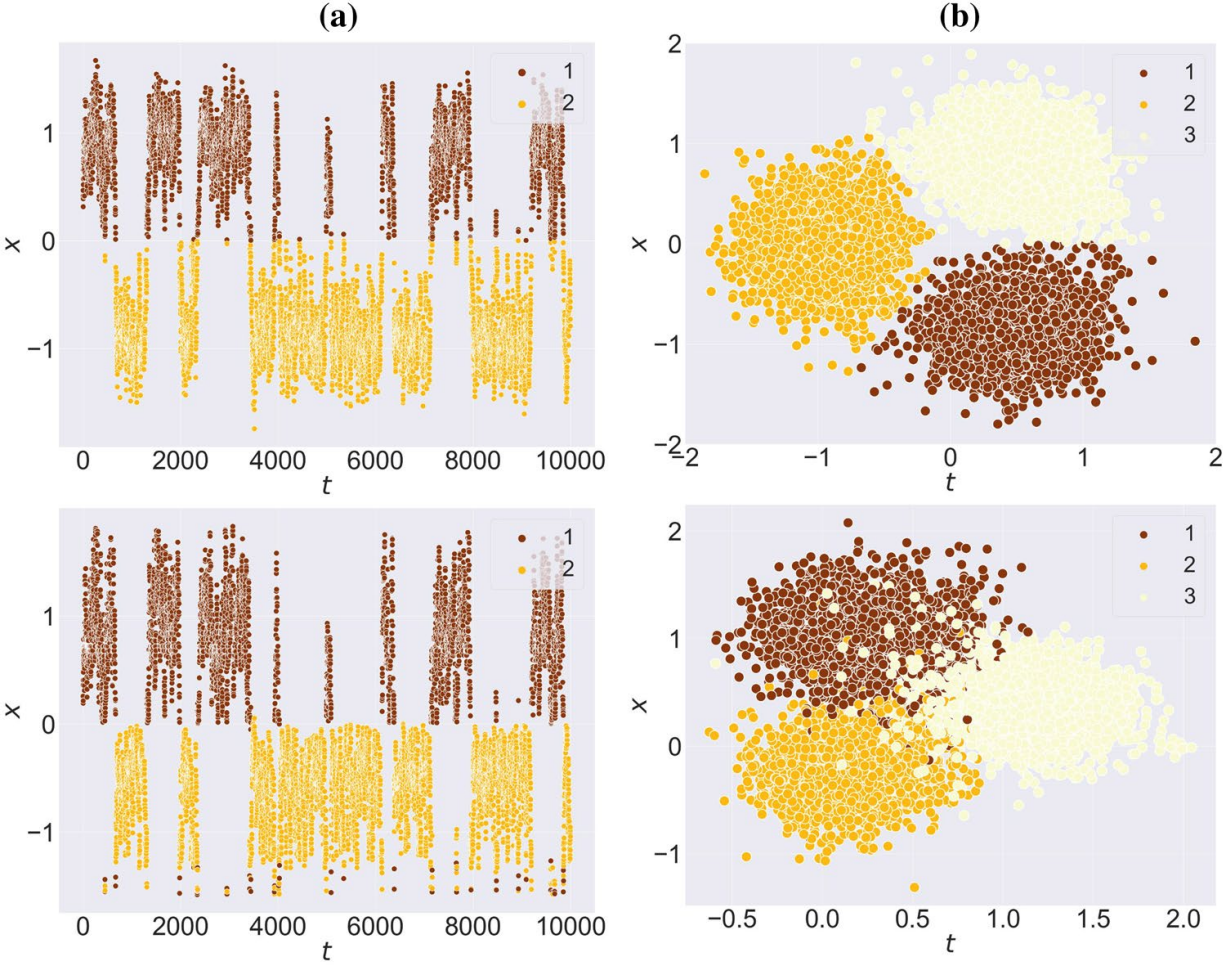
The comparison of an initial trajectory sampled from the SDE (1) (**top**) and the final graph embedding for (**bottom**): (**a**) the *npos_2WellGraph* dataset and (**b**) for the *pos_3WellGraph* dataset.

The evolution of the graph embedding during the training for both synthetic datasets — npos_2WellGraph and pos_3WellGraph — are illustrated in Fig. 2. The visualization demonstrates that during the training, our model tends to capture the underlying metastable structure in the time-evolving graph. Moreover, at the end of the training, we see that our model learns the graph embedding maintaining the initial metastable dynamics. In the case of the npos_2WellGraph dataset, there is no obvious split between the two metastable states, the reason is that the initial SDE trajectory has points that are located on the boundary between two metastable states. Furthermore, we compare the initial SDE trajectory and the final graph embedding obtained from our model with $d=1$ for npos_2WellGraph and with $d=2$ for pos_3WellGraph. The result for npos_2WellGraph is presented in Fig. 3a which shows that two trajectories are almost identical. The same result can be seen for pos_3WellGraph in Fig. 3b. These results indicate that the model is capable of extracting the underlying metastable dynamics in the time-evolving graph.

In Table 2, we can see that the results of visualization are reinforced by the high ARI values of our model. From the table can also be seen that the graphKKE method outperforms our model in the case of the pos_2WellGraph and pos_3WellGraph datasets. However, if we aim to have lower dimensionality of the graph embedding, then this method will fail to produce the same clustering accuracy. As the evidence for the visualization of the graph embedding obtained with graphKKE (Fig. 5), we see that graphKKE + PCA fails to produce a visualization with clear separated metastable states. Moreover, considering the results of other graph representation learning (Table 2), node2vec fails completely to learn the graph embedding of the time-evolving graph and graph2vec performs poorly on all synthetic datasets except npos_2WellGraph. It remains unclear whether graph2vec struggles to identify states in the positional data because states do not have unique topological patterns, or because this method is not meant to capture temporal changes.

**Table 2:** Adjusted Rand Index (ARI) for the comparative analysis on the graph clustering task. ARI close to 1 corresponds to greater accuracy in correctly identifying the ground truth states, and an ARI value close to 0 stands for random clustering.

Dataset	graphKKE	WL kernel	graph2vec	node2vec	PCA	our model
npos_2WellGraph	0.99	0.98	0.90	0.00	0.95	0.96
pos_2WellGraph	0.97	0.97	0.05	0.00	0.94	0.82
pos_3WellGraph	0.93	0.11	0.00	0.00	0.36	0.80
MovingPic	0.99	0.56	0.42	0.08	0.54	0.99
CholeraInf	0.88	0.65	0.29	−0.02	0.77	0.87

#### Result and discussion: real-world data

**Figure 4:**
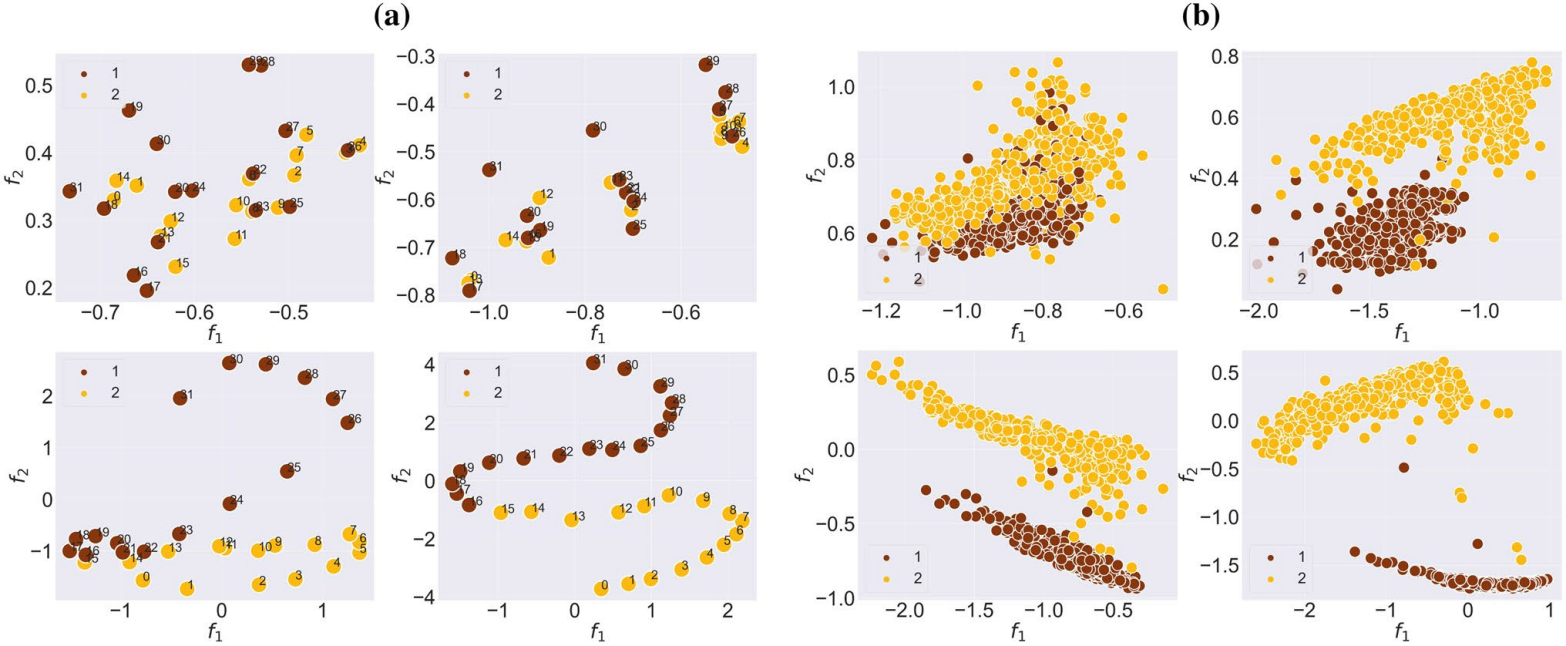
The evolution of the graph embedding of the time-evolving graph $\mathbb {G}$ during the training of our model on (**a**) CholeraInf and (**b**) MovingPic. The points are coloured according to ground-truth labels.

**Figure 5:**
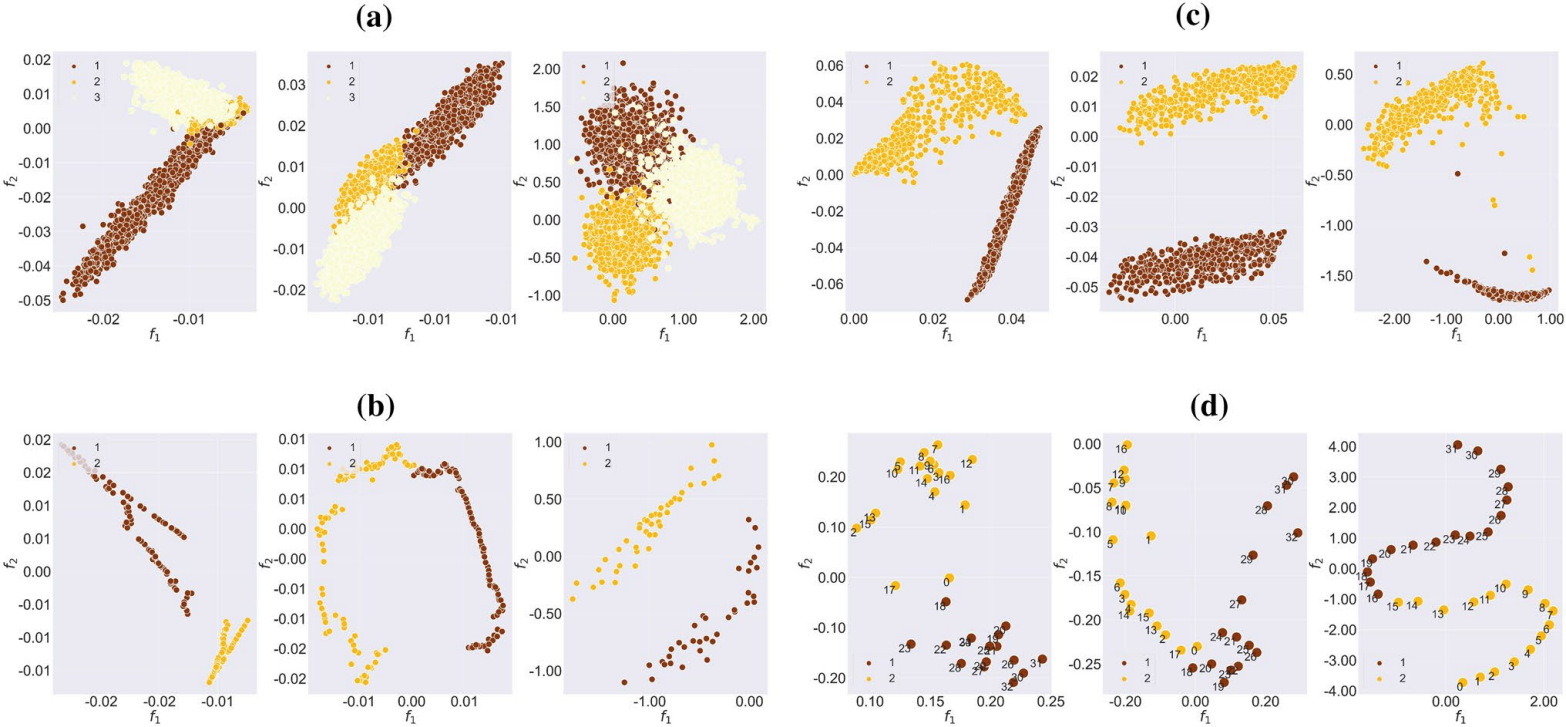
The graph embeddings of the time-evolving graph $\mathbb {G}$ for (**a**) npos_2WellGraph, (**b**) pos_3WellGraph, (**c**) MovingPic and (d) CholeraInf. From left to right: PCA on adjacency matrices, PCA on eigenfunctions of graphKKE and the result of our model.

In the case of real-world datasets, the evolution of the graph embedding during the training for MovingPic and CholeraInf are presented in Fig. 4. As it was in the case of synthetic datasets, our method is also able to identify the metastable behaviour in the time-evolving graph and preserve it in the new space. For CholeraInf we have added time points from the original dataset to see if the new space has the same time order as it was in the original high-dimensional space. If we compare the visualization of graph embedding from other methods, the result for MovingPic shown in Fig. 5c shows that all methods give relatively the same visualization. However, for the CholeraInf (Fig. 5d) our model preserves consecutive time points in the new space which indicates that one metastable state (healthy) follows other metastable states (ill).

The second part of this experiment aims at comparing our model with other dimensional reduction methods in the clustering task. Again from Table 2 it is evident that our model performs significantly better than WL kernel, graph2vec and node2vec. Node2vec performs poorly across all datasets, which is the result of a lower-order substructure embedding method meaning that it can model only local similarities and fails to learn global topological similarities.

#### Temperature parameter

**Figure 6:**
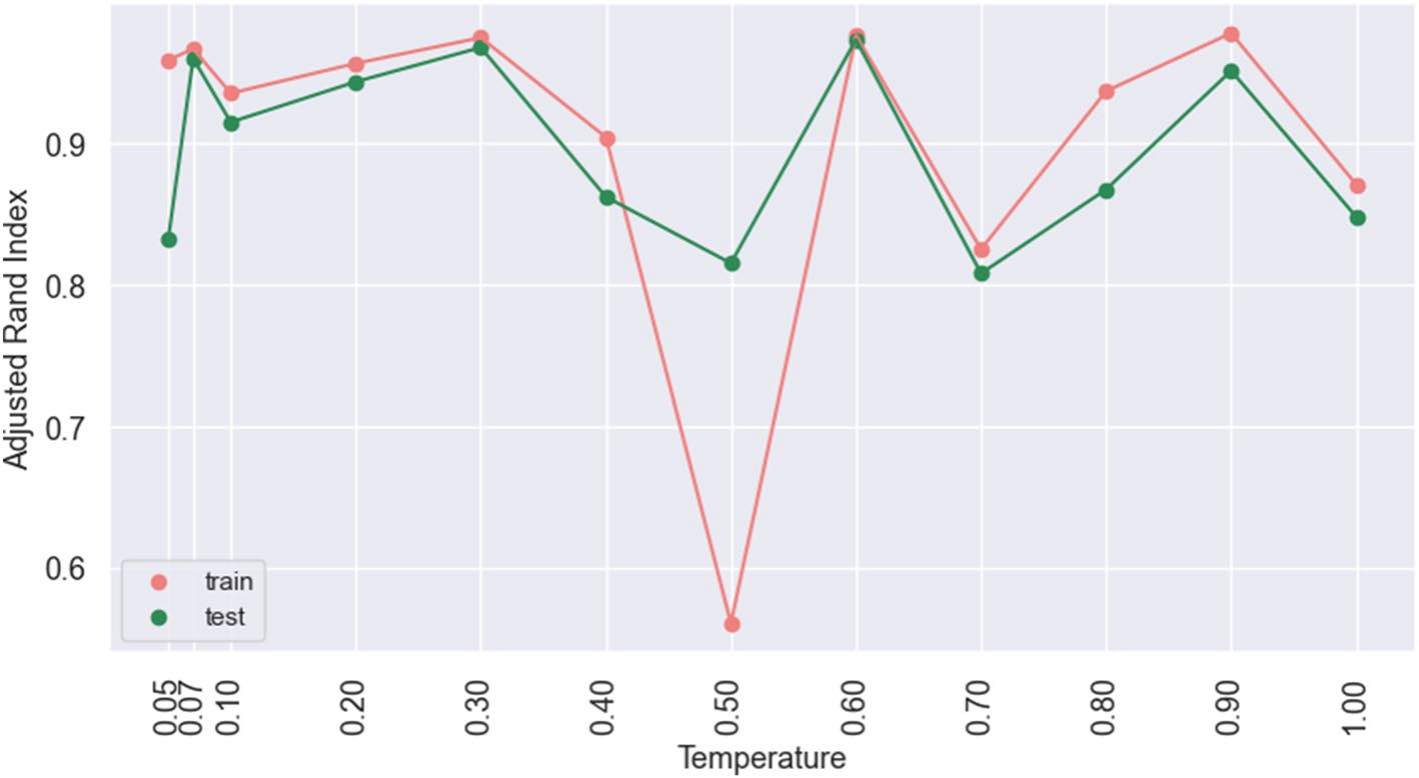
Adjusted Rand Index on the model trained on npos_2WellGraph with different temperature values of the contrastive loss. The red.

To understand the importance of the temperature $\tau$ in the contrastive loss, we train the model with different temperature values on npos_2WellGraph. The values between 0.05 and 1.0 have been chosen. According to Wang et al.^33^, the model with a small temperature tends to generate a more uniform distribution of graph embeddings and be less tolerant to similar samples. In our case, we have not noticed any dramatic changes in the performance. The ARI score for different temperature values can be seen in Fig. 6.

### Interpretability

An improved understanding of how the microbiome contributes to health and well-being can drive and accelerate the development of microbiome-based treatments. The most important question, which has not been answered yet, is which species or interactions of species are responsible for or affected by the changes which the microbiome undergoes from one state (healthy) to another state (diseased or antibiotic exposure). The presence of such valuable information can significantly improve modern treatments of various diseases. We assume that if it is feasible for the model to successfully find and discriminate metastable states, then there might be topological features in the time-evolving graph that make these metastable states different. Therefore, the main objective of this section is to provide insight into to which extent the model learns metastable states based on true discriminating topological features. And with regard to real-world data, we aim to find topological features of the time-evolving graph that make the two states, the cholera infection period and recovery period, different.

To achieve this, we will use an approach from Chefer et al.^34^ which is based on layer-wise relevance propagation (LRP)^35^. LRP is the family of explanation methods that leverages the layered structure of the network. It explains the prediction of a neural network classifier by backpropagating the neuron activation on the output layer to the previous layers until the input layer is reached.

The authors^34^ address the lack of the conservation property in the attention mechanism, which is an essential assumption of LRP and the numerical issues of the skip connections by applying a normalization to the computed relevance score. Moreover, they make use of the attention weights and propose to compute the final relevance scores by multiplying the relevance score of each Transformer layer with the gradient of the corresponding attention matrix summed up across the “head” dimension.

Unlike the original LRP and the approach mentioned in the last paragraph, where the decomposition starts from the classifier output corresponding to the target class, we have a similarity model that rather measures how similar a graph embedding of the time-snapshot graphs $G_t$ is to the embedding of the graph-snapshot $G_{t+1}$. For this reason, we start the redistribution from the layer where we compute the graph embedding $\hat{g}_t$ until the input layer is reached, and the final relevance is computed. We compute a relevance score for each time-snapshot graph in the test set. To obtain discriminating features of the whole state, we sum up relevance scores of time-snapshot graphs of each state.

#### Result and discussion: synthetic dataset

**Figure 7:**
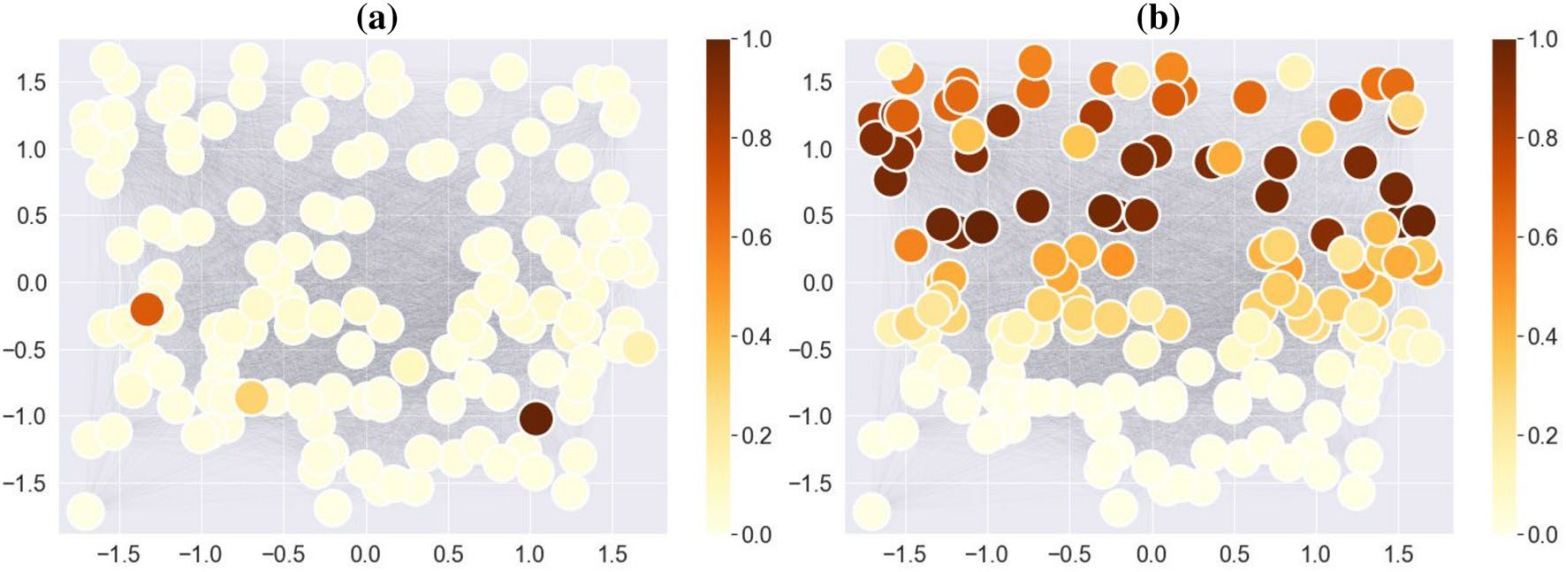
Fully-connected graphs for npos_2WellGraph dataset with nodes coloured based on the relevance scores of the LRP method that are summed across 2 states: (**a**) State 1 and (**b**) State 2. The locations of 2 states are obtained by clustering points of the graph embedding of the time-evolving graph via *k*-means.

We conduct this experiment on the npos_2WellGraph and the CholeraInf datasets. The result for npos_2WellGraph is demonstrated in Fig. 7. From the result, it is clear that the model can find topological features in the time-evolving graph that are unique for each metastable state. However, the interpretation of state 2 (Fig. 7b) highlights all nodes in the upper part of the time-snapshot graph, which is a true discriminating feature, whereas the interpretation of state 1 in turn shows only 4 nodes in the lower part of the time-snapshot graph. There is a necessity to mention that we have modelled the synthetic datasets in such a way that we know the location of nodes in the time-snapshot graphs. In the case of real-world datasets, we do not have the coordinates of nodes.

#### Result and discussion: real-world dataset

**Figure 8:**
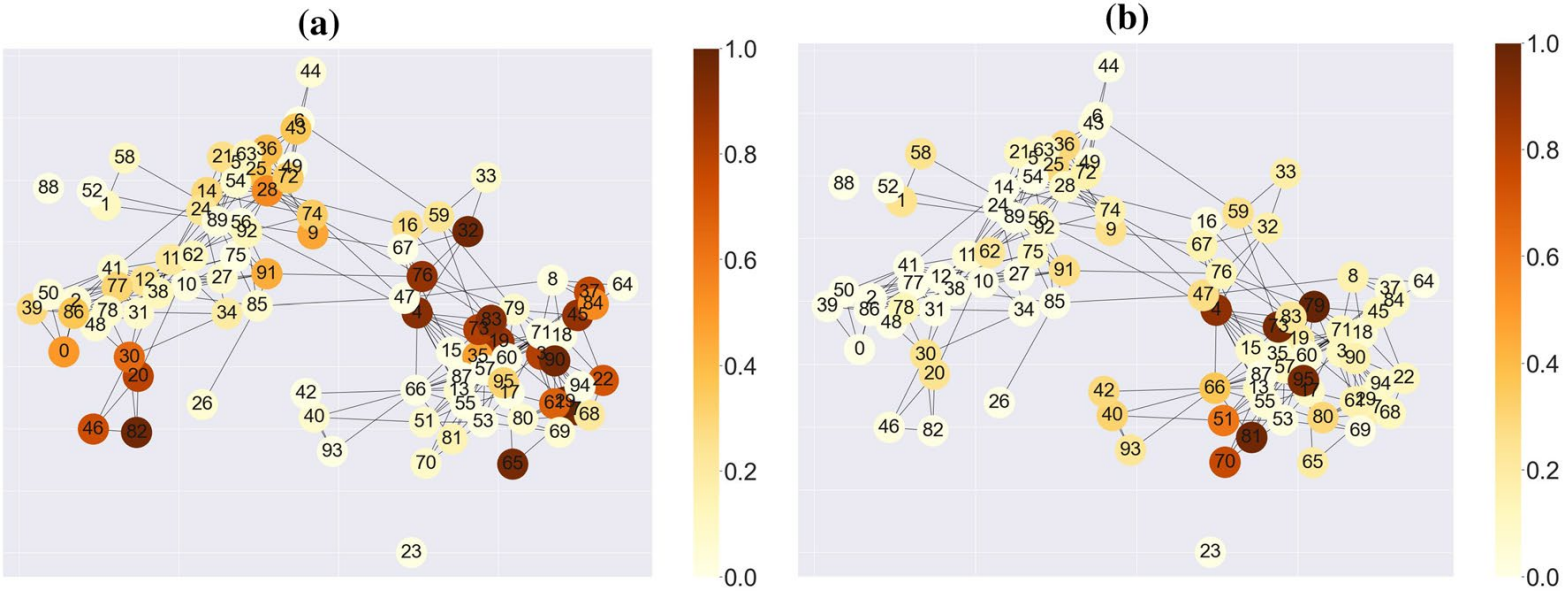
Co-occurrence interaction graphs of CholeraInf dataset with nodes coloured based on relevance scores of the LRP method, which are summed across each state: (**a**) diarrhea period and (**b**) recovery period. The locations of states are obtained by clustering points of the graph embedding of the time-evolving graph via *k*-means. The dark brown colour indicates nodes with the highest importance.

Here we focus on obtaining relevance scores for the real-world dataset, namely, CholeraInf. The result of LRP is presented in Fig. 8a for the diarrhea period (state 1) and in Fig. 8b for the recovery period (state 2). Both sub-figures show a correlation network of species that has been computed based on the OTU table (see more details about how this data has been pre-processed in Melnyk et al.^14^). The nodes represent species and the edges indicate the interactions between species. The colours encode the normalized relevance scores with higher values meaning the greater importance for the model and with lower values meaning the insignificance of nodes. If the colour of a node with the same label in both sub-figures is significantly different, this means that this node or interactions of this node with other nodes are discriminative features of metastable states. For example, the relevance score of node 82 is significant for state 1 (Fig. 8a) but not for state 2 (Fig. 8b).

Unlike the synthetic dataset, we do not know the ground truth discriminative features for this dataset. Further study of these results is needed to investigate if these interpretations have a biological meaning. For instance, there have been done numerous works^36^ that are mainly focused on statistical analysis to justify which bacteria/species are affected by, or on the contrary, cause shifts in microbiome compositions. Using the detected species from these works, we can compare them with the nodes that have shown the biggest impact on the model output.

## Discussion

We have presented a new approach that can simplify the analysis of time-evolving graphs with assumed metastability. Through an extensive set of experiments on both synthetic and real-world datasets, we have demonstrated that our approach is capable of projecting a time-evolving graph into a low-dimensional space retaining the metastable properties of the system. Moreover, we have illustrated one of the possible applications of this approach to microbiome data that enhances the analysis of metagenomic data in a way that takes into account a huge number of interactions among species. We have shown that by explaining the output of the model, we can find topological graph features, such as nodes or edges, that make the model arrive at a certain graph embedding. Concerning microbiome data, it means that our method coupled with a proper interpretation strategy can help to reveal underlying disease patterns in the data.

There are several directions for future work: 1) how to construct a time-evolving graph from metagenomic data such that it contains real dynamics occurring in the microbiome; 2) further biological analysis of results obtained from the interpretability of the model; 3) visualization of topological graph features, such as nodes and edges, that have impacted the model the most, and 4) mathematical explanation of how the model learns a graph embedding of the time-evolving graph maintaining metastable dynamics.

## Method

We first briefly introduce all necessary notations and definitions, which are used in the paper, and state the problem.

### Definitions

A graph *G* is a pair (*V*, *E*) with a non-empty set of nodes *V*(*G*) and a set of edges $E(G) = \{(v_i, v_j) \mid v_i, v_j \in V\}$. The set *V*(*G*) often represents the objects in the data and *E*(*G*) the relations between objects. We define *the adjacency matrix* of the graph *G* as the $n \times n$ matrix *A* with $A^{ij} = 1$ if the edge $(v_i, v_j) \in E(G)$, and 0 otherwise, where $n = \mid V \mid$.

Next, we define a metastability property, which was first mentioned in^14^. Consider a time-evolving graph $\mathbb {G}$ as a sequence of graphs $\mathbb {G} = (G_1,\dots , G_{T})$ at consecutive time points $\{1,\dots , T\}$ for some $T \in \mathbb {N}$, and $G_t$ being a time-snapshot of $\mathbb {G}$ at time *t*. The time-evolving graph $\mathbb {G}$ exhibits *metastable* behavior if $\mathbb {G}$ can be partitioned into *s* subsets $\mathbb {G} = \mathbb {G}_1 \cup \dots \cup \mathbb {G}_{s}$ for some $s \ll T$ such that for each time point $t \in \{1,\dots , T\}$ and $i, j = 1, \dots , s$, we have the following:

4 $$\begin{aligned} {\left\{ \begin{array}{ll} P(G_{t + 1} \in \mathbb {G}_i \mid G_t \in \mathbb {G}_j) \ll 1, \text { if } i \ne j\\ P(G_{t + 1} \in \mathbb {G}_i \mid G_t \in \mathbb {G}_j) \approx 1, \text { if } i = j, \end{array}\right. } \end{aligned}$$

where $P(\cdot )$ is a transition probability, and $\mathbb {G}_1, \dots , \mathbb {G}_{s}$ are called metastable states of the time-evolving graph $\mathbb {G}$, where *s* is the number of states.

### Problem statement

We define our problem as follows: *Given a time-evolving graph* $\mathbb {G} = (G_1, \dots , G_{T})$ *with assumed metastability property* (4),* we aim to represent each time-snapshot* $G_t$ *as a vector in a low-dimensional space *$\mathbb {R}^d$, *maintaining the metastable behaviour of *$\mathbb {G}$, *where d* *is a number of dimensions of the reduced space.*

In this section, we describe how we train the model to embed time-snapshot graphs into a low-dimensional space, maintaining the metastable behaviour of the graph. We first use the Transformer^13^ to compute the embedding of a time-snapshot graph. Further, we add a recurrent mechanism to the Transformer that facilitates the learning of temporal changes across consecutive time-snapshot graphs. Finally, we use contrastive learning to make representations of consecutive time-snapshots graphs, which share metastable behaviour, close.
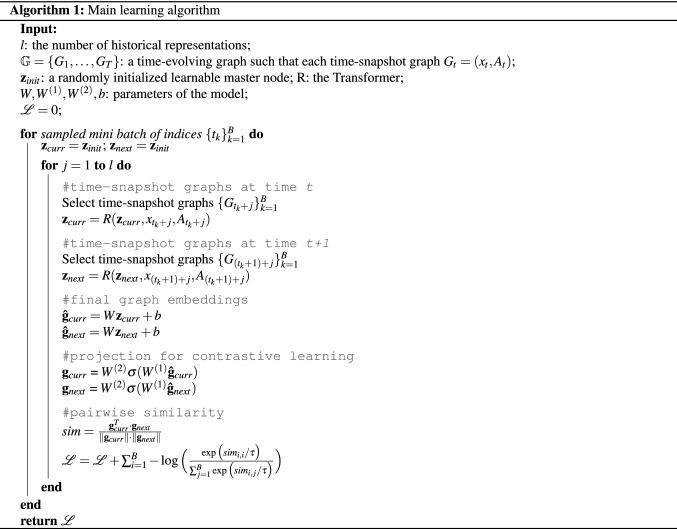


### Transformer

The Transformer is currently the state-of-the-art method in the field of NLP, where it has shown tremendous success in handling long-term sequential data. Recently, it has become a leading tool in other domains such as computer vision^37^ and graph representation learning^38,39^. We use the encoder part of the Transformer to learn node embeddings in each time-snapshot graph. The encoder has several stacked multi-head attention layers followed by a feed-forward layer. There is a residual connection around each of these two sub-layers that is also followed by a normalization layer.

Intuitively, the self-attention in time-snapshot graphs relates different nodes of the graph in order to compute a new representation of every node in the graph which we refer as a node embedding.

### Input

Let $\mathbb {G} = \{G_1,..., G_{T}\}$ be a time-evolving graph with node features $\{x_t^v\}_{v \in V(G_t)}$, $t = 1, \dots , T$. The input node features of each time-snapshot graph $G_t$ are embedded to $\textit{d}_m$-dimensional latent features via a linear projection and added to pre-computed node positional encodings. We demonstrate on two synthetic datasets with different topological structures that our model performs well in both cases: with positional information of nodes and without it. Moreover, in order to capture the topological structure of a single time-snapshot graph $G_t$, we feed an adjacency matrix $A_t$ to the Transformer as a mask, and we set attention weights to 0 whenever the corresponding adjacency matrix entries are 0.

### Model architecture

**Figure 9:**
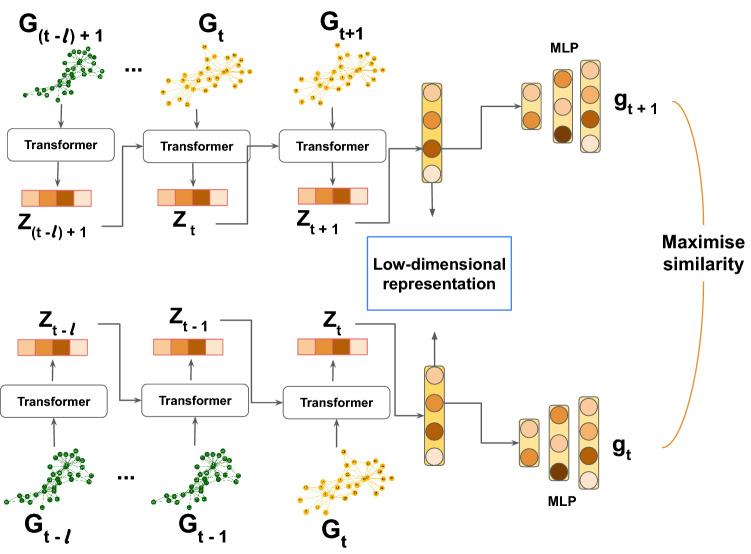
Overview of the method proposed in this paper.

Further, we explain important details of the training of the model. The overview of the model architecture can be found in Fig. 9.

Let $\mathscr {T} = \{t_k\}_{k=1}^{B}$ be a set of randomly sampled time points, and $\mathbb {G}_{\mathscr {T}} = \{G_{t_1}, \dots , G_{t_{B}}\}$ be a mini-batch of time-snapshots graphs sampled from $\mathbb {G}$ with a mini-batch size *B*. To facilitate the learning of temporal changes, we share the embedding of a time-snapshot graph with the consecutive time-snapshot graph in the temporal sequence. We define a master node that is connected to all nodes in the time-snapshot graph. Initially, the master node is represented as a learnable, randomly initialized vector. The Transformer computes the embedding of the master node, which we consider as a graph embedding. This graph embedding is then passed as the initial master node to the consecutive time-snapshot graph in the temporal sequence. We control the length of the temporal sequence with the hyperparameter *l*. Moreover, since we connect the master node with all other nodes in each time-snapshot graph, the size of the adjacency matrix changes, $A_t \in \mathbb {R}^{(n+1) \times (n+1)}$.

Formally, we update the graph embedding $z_t$ of the time-snapshot graph $G_t$ recursively as follows:

$$\begin{aligned} z_t = R(z_{t-1}, x_{t}, A_{t}), \end{aligned}$$

where *R* is the Transformer that updates node embeddings as discussed, $z_{t} \in \mathbb {R}^{ d_m}$ is a master node, $x_{t}$ is a vector of node features, and $A_{t} \in \mathbb {R}^{(n+1) \times (n+1)}$ is an adjacency matrix of $G_{t}$.

Finally, we project the graph embedding $z_{t} \in \mathbb {R}^{d_m}$ of the time-snapshot graph $G_{t}$ into the space with the dimension *d*, where a downstream task is defined. We denote the final embedding of the time-snapshot graph with $\hat{g}_{t} \in \mathbb {R}^d$:

$$\begin{aligned} \hat{g}_{t} = W z_{t} + b \end{aligned}$$

where *W* and *b* are learnable parameters. We use two hidden layers and a non-linear activation function in order to project the graph embedding $\hat{g}_{t}$ into the space, where the contrastive learning is defined, as it is done in Chen et al.^40^.

Furthermore, we explain how we use contrastive learning to make embeddings of consecutive time-snapshots graphs to preserve the metastable behaviour in the low-dimensional space.

### Contrastive learning

Intuitively, contrastive representation learning can be considered as learning by comparing. Thus, the goal is to find a low-dimensional space where samples from the same instance are pulled closer, and samples from different instances are pushed apart. Formally, given a vector of input samples $x_i, i=1, \dots , B$ with corresponding labels $y_i \in \{1, \dots , C\}$ among *C* classes, contrastive learning aims to learn a function $f_\theta (x)$ that can find the low-dimensional representation of *x* such that examples from the same class have similar representations, and samples from different classes are far away from each other in the new space. One always needs to have negative and positive samples to apply contrastive learning, For this reason, we make the following assumption.

*Assumption.* According to the definition of metastability (4), the probability of two consecutive time-snapshot graphs $G_{t}$ and $G_{t+1}$ being similar is almost 1 and so should be the probability for their graph embeddings $\hat{g}_{t}$ and $\hat{g}_{t+1}$.

In other words, we consider a pair of graph embeddings $(\hat{g}_{t}, \hat{g}_{t+1})$ as a positive pair and pairs $(\hat{g}_{t}, \hat{g}_{t+j})$ as negative pairs, where *j* is randomly sampled from $\{2, \dots , T\}$. It is feasible for negative samples to be of the same metastable state as the positive sample, but at different time points.

We use InfoNCE^41^ with $\hat{g}_{t+1}$ being the positive sample:

$$\begin{aligned} \mathscr {L} = -\log \frac{\exp {\big (\hat{g}_t \cdot \hat{g}_{t+1} / \tau \big )}}{\sum _{i=1}^B \exp {\big (\hat{g}_t \cdot \hat{g}_i / \tau \big )}}, \end{aligned}$$

where $\tau$ is a temperature hyperparameter and the similarity is measured by the dot product. The experiments from Wang et al.^33^ have shown that the temperature hyperparameter is important in controlling the local separation and global uniformity of the embedding distribution. Minimizing this loss function forces the parameters of the model to be tuned such that graph embeddings of two consecutive time-snapshot graphs are as close as possible. The detailed algorithm can be found in Algorithm 1.

### Positional encoding

Most graph neural networks learn structural node information that is invariant to the node positions. However, there are cases when topological information is not enough. To demonstrate this, we conduct experiments on two different synthetic datasets. The first data has metastable states with defined graph features that can be distinguished only by the position information of nodes. Each metastable state in the second data has specific graph features, which are easily distinguished with just topological information.

To incorporate the positional information, we use the same positional encoding as in Gehring et al.^42^:

$$\begin{aligned} p_{pos, 2i} = \sin {pos/10000^{2i/d_m}} \end{aligned}$$

$$\begin{aligned} p_{pos, 2i+1} = \cos {pos/10000^{2i/d_m}}, \end{aligned}$$

where *pos*, *i* and $d_m$ denote a position of the node in the time-snapshot graph, the dimension in the positional encoding and the dimension of node embedding, respectively.

Through a set of various experiments in the next section, we demonstrate on synthetic and real-world datasets that our method is capable of learning a graph embedding of the time-evolving graph.

## Data Availability

The datasets used and/or analysed during the current study are available from the corresponding author upon reasonable request.

## Code availability

Code can be found here: https://github.com/k-melnyk/deep-metastability.
